# A national evaluation of a dissemination and implementation initiative to enhance primary care practice capacity and improve cardiovascular disease care: the ESCALATES study protocol

**DOI:** 10.1186/s13012-016-0449-8

**Published:** 2016-06-29

**Authors:** Deborah J. Cohen, Bijal A. Balasubramanian, Leah Gordon, Miguel Marino, Sarah Ono, Leif I. Solberg, Benjamin F. Crabtree, Kurt C. Stange, Melinda Davis, William L. Miller, Laura J. Damschroder, K. John McConnell, John Creswell

**Affiliations:** 1 Department of Family Medicine, Oregon Health & Science University, 3181 SW Sam Jackson Park Road, Portland, OR 97239 USA; 2 Department of Medical Informatics and Clinical Epidemiology, Oregon Health & Science University, Portland, OR USA; 3 Department of Epidemiology, Human Genetics, and Environmental Sciences, University of Texas School of Public Health, Dallas Regional Campus, Dallas, TX USA; 4 Department of Veteran Affairs, Center to Improve Veteran Involvement in Care (CIVIC), VA Portland Health Care System, Portland, OR USA; 5 HealthPartners Institute, Minneapolis, MN USA; 6 Department of Family Medicine and Community Health, Rutgers-Robert Wood, Johnson Medical School, New Brunswick, NJ USA; 7 Departments of Family Medicine and Community Health, Epidemiology and Biostatistics, Sociology and the Case Comprehensive Cancer Center, and Clinical and Translational Science Collaborative, Case Western Reserve University, Cleveland, OH USA; 8 Oregon, Rural Practice-Based Research Network, Portland, OR USA; 9 Department of Family Medicine, Lehigh Valley Health Network, Allentown, PA USA; 10 Center for Clinical Management Research and PROVE QUERI, VA Ann Arbor Healthcare System, Ann Arbor, MI USA; 11 Center for Health Systems Effectiveness and Department of Emergency Medicine, Oregon Health and Science University, Portland, OR USA; 12 Department of Family Medicine, University of Michigan, Ann Arbor, MI USA

**Keywords:** Dissemination and implementation research, Primary care practice extension, Practice facilitation, Regional learning collaboratives, Primary care practice, Cardiovascular disease prevention, Practice capacity, Quality improvement, Multi-site evaluation

## Abstract

**Background:**

The Agency for Healthcare Research and Quality (AHRQ) launched the EvidenceNOW Initiative to rapidly disseminate and implement evidence-based cardiovascular disease (CVD) preventive care in smaller primary care practices. AHRQ funded eight grantees (seven regional Cooperatives and one independent national evaluation) to participate in EvidenceNOW. The national evaluation examines quality improvement efforts and outcomes for more than 1500 small primary care practices (restricted to those with fewer than ten physicians per clinic). Examples of external support include practice facilitation, expert consultation, performance feedback, and educational materials and activities. This paper describes the study protocol for the EvidenceNOW national evaluation, which is called Evaluating System Change to Advance Learning and Take Evidence to Scale (ESCALATES).

**Methods:**

This prospective observational study will examine the portfolio of EvidenceNOW Cooperatives using both qualitative and quantitative data. Qualitative data include: online implementation diaries, observation and interviews at Cooperatives and practices, and systematic assessment of context from the perspective of Cooperative team members. Quantitative data include: practice-level performance on clinical quality measures (aspirin prescribing, blood pressure and cholesterol control, and smoking cessation; ABCS) collected by Cooperatives from electronic health records (EHRs); practice and practice member surveys to assess practice capacity and other organizational and structural characteristics; and systematic tracking of intervention delivery. Quantitative, qualitative, and mixed methods analyses will be conducted to examine how Cooperatives organize to provide external support to practices, to compare effectiveness of the dissemination and implementation approaches they implement, and to examine how regional variations and other organization and contextual factors influence implementation and effectiveness.

**Discussion:**

ESCALATES is a national evaluation of an ambitious large-scale dissemination and implementation effort focused on transforming smaller primary care practices. Insights will help to inform the design of national health care practice extension systems aimed at supporting practice transformation efforts in the USA.

**Clinical Trial Registration:**

NCT02560428 (09/21/15)

## Background

The Agency for Healthcare Research and Quality (AHRQ) launched the EvidenceNOW Initiative in 2015 to promote delivery of evidence-based cardiovascular disease (CVD) preventive care in smaller primary care practices with limited resources and experience with quality improvement (QI), and to expand the nation’s capacity to rapidly translate evidence into practice. AHRQ funded seven regional Cooperatives spanning 12 states in the USA. Each Cooperative received a 3-year grant to create a health practice extension infrastructure to implement interventions aimed at decreasing cardiovascular risk for their patient populations within approximately 250 primary care practices, each with less than ten clinicians and with limited quality improvement capacity. Along with providing active external support, the Cooperatives will evaluate practice improvement efforts. In the call for proposals, AHRQ required use of measures that are common across the Cooperatives, including measures of CVD clinical quality and practice capacity outcomes. Cooperatives are required to share these data with a national evaluation team, which AHRQ funded separately.

This paper describes the study protocol for the EvidenceNOW national evaluation, called Evaluating System Change to Advance Learning and Take Evidence to Scale (ESCALATES) which aims to comprehensively evaluate the dissemination and implementation (D&I) approaches of the seven Cooperatives to generate overarching, cross-Cooperative findings. This work will inform future large-scale D&I efforts and expand the understanding of the characteristics of effective practice facilitation and regional extension services for practice improvement.

### QI in primary care practice

In hospitals and health systems with substantial resources, large QI initiatives have been shown to improve care quality by creating communities of learning that change behavior on a large scale [1–3]. The majority of people in the USA, however, receive care in smaller practices (with less than ten clinicians) [4] and without external support for change. More than half of primary care clinicians in the USA practice in a setting of five or fewer clinicians and about one-third in practices with less than two clinicians [5]. Smaller practices face substantial challenges in transforming their approach to care delivery, including greater time constraints, fewer staff, and fewer resources to support change than larger practices [6–10]. Few small practices have the time, resources, or expertise to develop QI capacity, and typically these practices need external support [11, 12] which may include performance feedback and benchmarking [13–15], practice facilitation or coaching [16–19], academic detailing or expert consultation [20–25], and participating in learning collaboratives [26–29]. In the context of the rapid pace of practice transformation in the USA, primary care practices, particularly smaller ones, can become overwhelmed and experience change fatigue, which further slows QI progress by creating tension and conflict, burnout, turnover, and resistance to change [10]. External organizations may be able to assist practices with QI, and improve adherence to evidence-based guidelines [11]. One type of external organization that might help with this is the practice care health extension [30, 31]. The concept of extension is dynamic and the most commonly recognized extension system is a rural or agricultural extension as seen in the USA and internationally. The practice extension, like the agricultural extension, might use an administrative structure, in partnership with local communities, to bring about change in practices through a combination of education, problem solving support, technical advice, and resources to help practices make improvements and proactively respond to future developments.

### Cardiovascular disease and the ABCS

Many people do not receive guideline-concordant health care. This is true even for relatively low cost treatments such as aspirin prescribing, blood pressure and cholesterol control, and smoking cessation (the ABCS), each of which can significantly reduce risk of cardiovascular disease (CVD) [32, 33]. CVD is the leading cause of death in the USA, with stroke or heart disease contributing to one out of every three deaths [34]. Primary risk factors for CVD (high blood pressure, high cholesterol, smoking, obesity) are often preventable or treatable [34]. While increasing rates of ABCS is known to significantly reduce risk for CVD, the overall uptake of these effective preventive services is low, even among a clinic’s high-risk patients [32, 33]. Nationally, only 53 % of people with documented hypertension have blood pressures at or below target levels [34], only one-third of people with elevated cholesterol are adequately managed by, e.g., taking a statin, and less than 25 % of the individuals who smoke receive assistance with quitting.

A recent study on pay-for-performance incentives in small practices with electronic health records (EHRs) found that baseline ABCS rates were low in small primary care practices for aspirin therapy, blood pressure control, and smoking cessation [35]—a situation that is even worse among clinics caring for the poor and for racial/ethnic minority populations [36, 37]. If the ABCS services were consistently delivered in primary care, the burden of CVD would be greatly reduced [38, 39]. To address this quality gap, in 2011, the Department of Health and Human Services (DHHS) and the Center for Disease Control and Prevention (CDC) launched the Million Hearts Initiative for Medicare and Medicaid Services (CMS) members/patients. It seeks to prevent one million heart attacks and strokes by 2017 by empowering individuals to make healthy choices and by improving delivery of ABCS services [32, 33]. The EvidenceNOW initiative is focused on improving the delivery of ABCS services among a large cohort of smaller primary care practices.

As the national evaluation of EvidenceNOW, ESCALATES will accomplish the following aims:

1. Evaluate the overall EvidenceNOW initiative by engaging regional Cooperatives in harmonizing measures, working together to collect similar qualitative and quantitative data, identifying lessons learned, and fostering rapid-cycle learning;
2. Identify practice, organization, and contextual factors associated with meeting ABCS performance goals at baseline (prior to intervention);
3. Identify the intervention strategies that are most effective in improving ABCS performance goals/targets and practice capacity in relation to practice, organization, and contextual factors, and learn why some strategies are more effective by integrating qualitative and quantitative data; and
4. Engage, rapidly disseminate, and evaluate the impact of disseminating actionable findings to key external stakeholders.

The ESCALATES evaluation is a collaboration among investigators at Oregon Health and Science University, University of Texas School of Public Health, Rutgers Robert Wood Johnson Medical School, HealthPartners, Case Western Reserve University, University of Michigan, and Lehigh Valley Health Network.

## Methods/design

### Study design

ESCALATES is a prospective mixed methods observational comparative study using a range of qualitative and quantitative data collection approaches to accomplish the study aims (see Table 1). The ESCALATES study is informed by the Learning Evaluation approach in that we will analyze clinical relevant outcome measures that do double-duty as performance measures for practices, thereby fostering learning for practices; we collect and analyze real-time quantitative and qualitative data on important contextual factors, and the goal is to foster cross-Cooperative learning from process and outcome data [40]. Table 2 list key elements of the evaluation.

**Table 1:** Data Sources

	Description	Level	Source of data	Type of data	Frequency
Cooperative grant proposals	Cooperative’s initial plan for implementation	Cooperative-level	Collected by ESCALATES team with Cooperatives assistance	Qualitative	Pre-grant award
Other documents	Documents Cooperative’s develop (ex: recruitment materials, PF curricula, etc.)	Cooperative-level	Collected by ESCALATES team with Cooperatives assistance	Qualitative	Throughout study period
Online diaries	Online journal; approximately 5-13 people per Cooperative; document implementation experiences	Cooperative-level	Online, interactive communication platform	Qualitative	Post 2x mo.; Start-up to post-implementation
Cooperative site visits	To observe and understand how interventions are implemented	Cooperative-level	Fieldnotes	Qualitative	Annually years 2 and 3
Sustainability site visits	To understand how/which parts of the D&I infrastructure is sustained beyond the life of the grant	Cooperative-level	Fieldnotes	Qualitative	Year 4
Semi-structured interviews	To understand barriers/facilitators of specific aspects of implementation; experience with intervention; mechanisms of change	Cooperative-level	Interviews with Cooperative key stakeholders	Qualitative	Annually with 5-8 people or as needed
Clinical quality measures (ABCS)	Proportion of patients meeting ABCS and data source (e.g. EHR, chart review)	Aggregated by Practice	Medical Record (EHR extraction/chart reviews); collected by Cooperatives; shared with ESCALATES	Quantitative	Baseline through end of study; Quarterly
Stratified ABCS measures	Proportion of patients stratified by gender, age, race, ethnicity, insurance type meeting ABCS	Aggregated by practice	Medical record (EHR extraction); collected by Cooperatives; shared with ESCALATES	Quantitative	Baseline through end of study; Quarterly
Practice survey	Practice capacity (CPCQ), EHR adoption, practice demographics (internal and external characteristics)	Practice-level	Survey (online/paper); completed by office manager or designated clinic leader; collected by Cooperatives; shared with ESCALATES	Quantitative	Baseline, post-intervention, 6 m follow up
Practice member survey	Practice capacity (AR), focus on patients’ needs and resources, practice readiness to change, burnout, clinician attitudes towards new guidelines	Aggregated by Practice	Survey (online/paper) completed by a majority of practice members (target > 70 %); collected by Cooperatives; shared with ESCALATES	Quantitative	Baseline, post-intervention, 6 m follow up
External support intervention tracking	Type of external support provided, date of contact, mode of contact, duration of contact, practice engagement assessment	Practice-level	Collected by Cooperatives; shared with ESCALATES	Quantitative	Regular intervals; TBD by grantees and evaluation
Practice implementation intervention tracking	Strategies in place to improve ABCS, assessment of the extent to which strategies are implemented	Practice-level	Collected by Cooperatives; shared with ESCALATES	Quantitative	At least baseline and post-intervention
Practice site visits	To observe and understand how interventions are implemented	Cooperative-level	Fieldnotes	Qualitative	60 practice site visits across years 2 and 3
Patient pathways	To observe patients exposure to/experience with intervention	Practice-level	Observation by evaluation team during site visits	Qualitative	Observe five–ten patient visits at each practice visit
Context assessment	To understand the contextual factors of implementation	Cooperative-level	Interviews with Cooperative key stakeholders	Qualitative	Annually with two–three people or as needed

**Table 2:** Key elements of the ESCALATES evaluation

- Multi-level focus on Cooperatives and practices - Longitudinal data collection over three years - Baseline and quarterly data collection on cardiovascular measures - Collection of practice capacity outcome at baseline, and two time points post-intervention - Extensive qualitative data collection through document review, interviews, online diaries, and observation - Detailed external support and practice implementation tracking - Measurement of implementation science conceptual markers - Assessments of change in small, medium-sized family medicine practices - Formative and summative evaluation assessments - Mixed methods integration of the quantitative and qualitative results	

### Study setting and population

EvidenceNOW Cooperatives will support practices in 12 US states and are each partnering with local collaborators (e.g., regional extension centers, quality improvement organizations) to engage smaller primary care practices in their regions. At the Cooperative level, the study sample includes the following regions: Midwest: Illinois, Indiana and Wisconsin (Principal Investigator (PI): Kho); New York City (PI: Shelley); North Carolina (PI: Cykert); Northwest: Washington, Oregon and Idaho (PI: Parchman); Oklahoma (PI, Duffy); Southwest: Colorado and New Mexico (PI: Dickinson); and Virginia (PI: Kuzel). The ESCALATES evaluation will include the approximately 1,500 primary care practices, their staff members, and patients across the Cooperatives.

### Evaluation framework

The ESCALATES evaluation uses the Consolidated Framework for Implementation Research (CFIR) [41] and the Practice Change Model (PCM) to guide the study design [42]. The CFIR is a highly organized explanatory framework that identifies factors that potentially influence implementation success, organized across five domains: characteristics of the intervention (e.g., complexity); outer setting (e.g., external policies and incentives); inner setting (e.g., leadership engagement, compatibility); characteristics of individuals (e.g., knowledge and beliefs); and process (e.g., planning). The PCM is an empirically-derived, dynamic conceptual model for how change happens in primary care practices. The PCM complements and extends the CFIR by differentiating motivation and capacity or resources for change, emphasizing the importance of interdependencies that manifest among the contextual and environmental factors influencing intervention effectiveness, and expanding the CFIR concept of “inner setting” through delineation of the factors that shape motivation. Together, the PCM and CFIR provide a robust set of frameworks for evaluating the array of factors which directly inform measurement and that may influence the D&I interventions used by the Cooperatives.

### Data collection

In the ESCALATES evaluation, data will be collected at the Cooperative and practice levels. Table 2 shows the sources of data and timelines for data collection over this 4-year study.

### Cooperative-level data collection

#### Collection of documents

The ESCALATES team will collect Cooperative documents that are important to understanding the interventions they propose, the modifications they make to their plans, and the partnerships they develop to carry out their work. This includes, but is not limited to, grant applications, documents that establish changes to study and intervention design, training materials for Cooperative staff, meeting agendas and minutes, and educational materials for practices.

#### Online diaries

Each Cooperative will identify at least five diary-keepers who will participate in an online diary to document experiences during project development and evolution, including practice engagement, recruitment, and providing external support for implementation. The use of online diaries as a qualitative data collection method has been described in detail elsewhere [43]. Briefly, each team will have a private online diary space to post entries in a blog type format. Cooperatives will be encouraged to make diary entries at least twice a month. Only diary-keepers at a particular Cooperative and evaluation team members will be able to view entries from that Cooperative, and we shall maintain strict external confidentiality about the information posted within each Cooperative. The ESCALATES team will read entries weekly and interact with Cooperative diary-keepers via the online diary to encourage frequent posting.

#### Site visits

The ESCALATES team will visit each Cooperative annually to facilitate collaborative work and to fully understand the D&I approaches they are implementing and their lessons learned. The site visits will last approximately 2 days each year, and ESCALATES team members will meet with each Cooperative’s project personnel including the research team, practice facilitators, coaches, consultants, and other partners. The ESCALATES team will observe Cooperative intervention strategies, foster discussions on implementation experiences, and observe interactions among partnering institutions and with individual practices. In year 4 of the study, after funding for the Cooperatives has ended, the ESCALATES team will conduct an additional site visit that will focus on assessing the sustainability of the network infrastructure.

#### Cooperative semi-structured interviews

During site visits, the ESCALATES team will conduct five–ten semi-structured interviews with key stakeholders from the Cooperative. Interviews will focus on filling knowledge gaps about the intervention strategies being tested, understanding the experiences of partnering stakeholders, the mechanisms by which interventions are believed to change outcomes, and the factors that affect or explain implementation experiences. Interviews will follow a guide to be refined for use with each Cooperative and each key stakeholder. Interviews will last 40–60 min and will be audio recorded and professionally transcribed.

### Practice-level data collection

The ESCALATES team will work with Cooperatives to harmonize collection of key practice-level outcome measures required by AHRQ (e.g., ABCS measures, practice capacity). As described below, ABCS data collection will require extraction from practices’ EHRs and practice capacity measures will require self-report or survey data collection methods. The ESCALATES team will also engage Cooperatives in a collaborative process to prioritize and harmonize the collection of additional measures that align with our goals.

#### ABCS data

Cooperatives will provide practice-level ABCS data to the ESCALATES team, as specified in Table 3. Cooperatives will collect data from participating practices’ EHRs using a range of data extraction methods (e.g., programming, manual chart review). ABCS measures will be collected at baseline—before interventions begin—and quarterly through the end of each Cooperative’s study. Cooperatives will also contribute practice-level ABCS data stratified by gender, race, ethnicity, age, and insurance type to allow for the examination of disparities.

**Table 3:** Clinical quality outcome measures

Measure	Description (CMS e-quality measure, National Quality Forum measure)	Source of data
Proportion of patients in a practice at risk for CVD receiving guideline-concordant care (ABCS) Data will be reported for the practice overall, and stratified by gender, race, ethnicity, age, and insurance type.	Patients within each practice who are: 18 years of age and older who were discharged alive for acute myocardial infarction, coronary artery bypass graft or percutaneous coronary interventions in the 12 months prior to the measurement period, or who had an active diagnosis of ischemic vascular disease during the measurement period, and who had documentation of use of aspirin or another antithrombotic during the measurement period (Aspirin, A, CMS164v4, NQF0068)	Medical Record (EHR extraction/chart reviews); collected by Cooperatives; shared with ESCALATES
	18-85 years of age who had a diagnosis of hypertension and whose blood pressure was adequately controlled (<140/90 mmHg) during the measurement period (Blood Pressure, B, CMS165v4, NQF0018)	
	High-risk adult patients aged > = 21 years who were previously diagnosed with or currently have an active diagnosis of clinical atherosclerotic cardiovascular disease; OR adult patients aged > =21 years with a fasting or direct Low-Density Lipoprotein Cholesterol (LDL-C) level > = 190 mg/dL; OR patients aged 40-75 years with a diagnosis of diabetes with a fasting or direct LDL-C level of 70-189 mg/dL; who were prescribed or are already on statin medication therapy during the measurement year (Cholesterol Management, C, CMS347)	
	18 years and older, who were screened for tobacco use 1or more times within 24 months AND who received cessation counseling intervention if identified as a tobacco user (Smoking, S, CMS138v4, NQF0028)	

*ABCS data will be collected from baseline through end of Cooperatives' three-year study. Cooperatives will share data with our ESCALATES team quarterly

#### Practice surveys

In addition to ABCS clinical quality measures, information about practice characteristics is needed as well as practice capacity for change, a key outcome measure. Each Cooperative will administer surveys to collect practice level data on characteristics and capacity using descriptive questions. The “practice survey” (PS), will collect data on practice organization and infrastructure (e.g., size, ownership, staffing, EHR capacity). This survey will include the change process capacity questionnaire (CPCQ) [44, 45], which is a measure of both a practice’s capacity for QI and the strategies used to improve. One person in the practice (preferably a practice leader) will complete the PS. The second survey, the “practice member questionnaire” (PMQ), will be administered to practice members and include a measure of organizational culture and capacity using the adaptive reserve [10, 46–49] scale. In addition, the ESCALATES team will work with Cooperatives to identify high-priority measures to understand implementation, as informed by the CFIR and PCM frameworks. The PS and PMQ surveys will be administered by Cooperatives at baseline, immediately post-intervention, and 6-month post-intervention. The ESCALATES team will work with Cooperatives to help ensure a high response rate.

#### External support and practice implementation tracking

It is important for this evaluation to track characteristics of the interventions delivered to practices, including the type and intensity of external support interventions received, along with changes that practices implement to increase ABCS delivery. The ESCALATES team will work with Cooperatives to establish methods (e.g., practice facilitator contact logs, collaborative (onsite) or webinar attendance records) to track the type, frequency, duration and mode of external support provided to practices and their engagement with it. Data collection related to external support will continue throughout each Cooperative’s active intervention period, which will vary by Cooperative. The ESCALATES team will also work with Cooperatives to develop mutually agreeable methods to track and share the change strategies practices implement to improve ABCS (e.g., registries, standing orders) and the extent to which these changes are implemented within these practices.

#### Practice site visits

The ESCALATES team will also conduct practice site visits. We will purposively select a sample of 40–60 practices using both qualitative and quantitative data to do so. The ESCALATES team will develop a matrix that ranks practices by ABCS outcomes at baseline (from high to low), and will also include percent change in ABCS over study period, Cooperative, ownership, and characteristics relevant to the region within the Cooperative (e.g., rural, underserved) where relevant. The ESCALATES team will use this matrix of quantitative information along with qualitative implementation data to select a maximum variation sample of high and low performing practices, approximately six–eight per Cooperative. We will conduct preliminary analyses based on the first two–three visits and use this information to revise sample selection criteria as needed. This iterative process of selecting practices, collecting data, and using this information to refine sampling decisions will continue until variations in outcomes are adequately explained as indicated by theme saturation.

The ESCALATES team will work with Cooperative teams to invite practices to participate. Practices will receive a monetary fee for their time and participation. A multi-person team will spend 1–2 days in a practice intensively observing how ABCS care is delivered and conduct interviews (scheduled to minimize disruption) with practice members to understand their experiences with external support and their efforts to implement interventions aimed at increasing delivery of ABCS. Interviews will also identify practice, organization and contextual factors influencing implementation. Interviews guides and observation templates will be refined and tailored to each practice and to each member based on information from online diaries, Cooperatives, member role, and site visit preparation calls with practice members.

#### Patient pathways

At each site visit, study staff will conduct five–ten patient pathways [50]. Patient pathways are designed to observe patients’ exposures to and experiences with how the practice has implemented changes. Front desk staff will alert a field researcher when a patient meeting sampling criteria checks-in. The field researcher will ask the doctor and patient for permission to observe the visit. If both agree, the field researcher will follow the patient through the visit (e.g., intake, visit with clinician, check-out), being unobtrusive and making brief notes to be written up later as fieldnotes.

### Analytic strategy

This study aims to answer the following research questions:

*Cross-sectional research questions (baseline):*

1. What are the rates of meeting ABCS performance goals/targets among a large sample (>1500) of small- and medium-size primary care practices?
2. What are the practice, organizational and contextual factors associated with varying degrees of meeting ABCS performance goals/targets at baseline (prior to implementing any intervention)?

*Longitudinal Research Questions:*

1. What are the types of intervention strategies (e.g. receiving different external support) implemented by Cooperatives, and how do they develop infrastructure for delivering these strategies to practices?
2. Across practices, what are the effects of intervention strategies on change in practice processes and ABCS performance measures and practice capacity (e.g., CPCQ, adaptive reserve) over time, and how do these intervention strategies interact with practice, organization, and contextual factors to explain the observed change?
3. Across practices, why are some intervention strategies more effective than others?
4. What are the explanations for variation in outcomes across Cooperatives on ABCS and practice capacity outcomes (e.g., how do high and low performing practices differ, and how do intervention strategies and intensity, practice and organization characteristics contribute to these differences)?

Below, we describe the analytic strategy we will use to answer study research questions:

### Cross-sectional research questions (baseline)

At baseline (pre-intervention), the ESCALATES team will determine the overall rates of meeting ABCS performance targets across Cooperatives. These data will provide nationally representative estimates of ABCS delivery rates among small- to medium-size primary care practices, identify the current status of ABCS target attainment, and determine how much room for improvement is available over the study period.

The ESCALATES team will then evaluate the practice, organizational and contextual factors associated with ABCS performance prior to implementing an intervention. Initial analyses will be descriptive, using data visualization methods. The principal outcome of interest is the proportion of eligible patients meeting ABCS performance goals for each practice (i.e., continuous values from 0 to 1). We will perform univariable and multivariable beta regressions with variable dispersion [51]. Beta regression models are common in analysis of rates and proportions. We will use survey measures informed by the CFIR and PCM to construct independent variable selection models that help to identify relevant variables among many potential candidates to achieve better interpretability and uncover previously unknown relationships in the data. Variable selection for the proposed beta regression model will be performed using a regularization technique [52] similar to the adaptive LASSO (regression analysis). The ESCALATES team will select the final model based on the Bayesian information criterion (BIC) by choosing the tuning parameter with the lowest BIC value because regularization-type methods with a BIC-type tuning parameter selector, under certain criteria, have been shown to do consistent variable selection [53].

### Longitudinal research questions

#### Identifying and distinguishing Cooperatives’ intervention approaches

A central aspect of Cooperatives’ interventions involves developing the infrastructure needed to rapidly deliver external support to approximately 250 practices. The ESCALATES team will analyze qualitative data to characterize the specifics of each Cooperative’s external support strategy and to create typologies that distinguish interventions and characterize the infrastructure Cooperatives develop to support practices. Typologies are a system for categorizing Cooperatives and their participating practices into groups based on a set of salient characteristics. Groupings will be quantified and become variables in subsequent quantitative analyses. This mixed methods procedure is to converge the data using data transformation so that a quantitative analysis can be used based on quantifying the qualitative findings. To do this, the ESCALATES team will analyze qualitative data using a grounded theory approach [54–56] to characterize the factors that emerge as barriers/facilitators to implementation (e.g., practice, context/environmental, and intervention characteristics), how and by whom external support is delivered and the factors that affect this, and what changes practices implement and why. We will make comparisons across Cooperatives, paying particular attention to factors leading to a Cooperative excelling or struggling in response to the intervention. We will create matrices to display data for comparative analyses as suggested by Miles and Huberman [57]. As the ESCALATES team develops initial classifications, these will be shared with Cooperatives as a form of “member checking,” a qualitative research verification step accomplished by asking key informants to validate study findings [58–61]. This analytic strategy will result in rich interpretive summaries of each Cooperative’s intervention, particularly with respect to core features of intervention strategies and other characteristics the ESCALATES team identify as important to D&I. Summaries and matrices will lead to the development of a series of typologies that categorize and transform important aspects of the Cooperatives and their interventions into quantitative variables. For example, external support (e.g., practice facilitation, expert consultation), mode of intervention (e.g., face-to-face, email, phone), frequency (number) and duration (length) of contacts might be made into a composite variable to represent intensity of external support for use in subsequent quantitative analyses.

#### Effect of intervention strategies on change in outcomes

Examining the effectiveness of external support interventions will involve estimating differences in ABCS and practice capacity outcomes for different intervention strategies received by practices engaged in each Cooperative, as well as differences in practice implementation. Intervention strategies will be compared to each other and/or to a control group of practices using a differences-in-differences (DID) approach [62]. The DID model is a flexible approach which can accommodate different study designs Cooperatives may propose to evaluate their own interventions. Conceptually, cluster-randomized designs, interrupted time studies, and step-wedge designs can be nested within the DID framework. The primary requirements are the inclusion of baseline (pre-intervention) data and data from a control group. In addition to examining the effectiveness of intervention typologies as assigned, we will also conduct sub-analyses that include differing levels of fidelity to quality improving strategies practices use. The DID approach has been frequently used by health services researchers and economists to account for potential secular effects and changing policies that would affect both intervention and control groups over time, while adjusting for potential confounders [7, 63–68].

The overall analysis to assess intervention strategies on ABCS performance can be considered as shown in the equation

$$ {\boldsymbol{Y}}_{\boldsymbol{it}}=\boldsymbol{f}\left(\boldsymbol{\alpha} +{\displaystyle \sum_{\boldsymbol{j}=1}^{\boldsymbol{r}}}{\boldsymbol{\beta}}_{\boldsymbol{j}}{\boldsymbol{I}}_{\boldsymbol{j}}+\boldsymbol{\delta} {\boldsymbol{\tau}}_{\boldsymbol{j}}+{\displaystyle \sum_{\boldsymbol{j}=1}^{\boldsymbol{r}}}{\boldsymbol{\theta}}_{\boldsymbol{j}}{\boldsymbol{I}}_{\boldsymbol{j}}{\boldsymbol{\tau}}_{\boldsymbol{j}}\right), $$

where: ***Y***_***it***_ represents clinic *i’*s performance on an outcome measure (i.e., one of the ABCS measures, mean adaptive reserve score) at time *t*,; ***I***_***j***_ represents a dummy variable (intervention strategy received variable described above) taking a value of 1 if the clinic was involved in intervention *j* and zero otherwise; and ***τ***_***j***_ is a dummy variable taking a value of 1 if the observation took place after the intervention and 0 otherwise, and *f* represents a general function that captures our intent to model performance through a beta regression for ABCS measures and linear regression for practice capacity outcomes. The coefficient ***α*** is an overall intercept representing average baseline performance for all clinics; ***β***_***j***_ represents the incremental baseline performance differential between clinics in typology *j*, and ***δ*** represents the change in performance pre- and post-intervention, for all clinics. The ***θ***_***j***_ coefficients are the coefficients of interest, as they capture the relative performance improvement for each group using intervention typology *r*. The qualitative analyses described above will produce *r* variables (e.g., high, medium, low intensity support strategy) (*r* lying somewhere between 3 and 6). In this model, control groups serve as the reference. This equation can be adapted to include additional dummy variables for each time period or a continuous variable to capture linear time trends as well as fixed effects (equivalent to dummy variables) for practices.

The ESCALATES team will use two-way fixed effects models (i.e., a fixed effect for each practice and for each time period) so the results do not rest on some of the stricter assumptions required for random effects models. However, we will also conduct analyses by introducing a practice random intercept and slope to allow intervention effects to vary across practices in the different study arms. This approach explicitly models different clustering levels (e.g. Cooperative, region, practice) [63]. In this case, rather than treating the practice as a nuisance factor, this approach allows each practice to respond differently over time to the intervention and allows the ESCALATES team to estimate those changes and study the heterogeneity of effects due to variation in context across Cooperatives and differences across practices within each Cooperative.

The analyses described above will be conducted for each ABCS measure. In addition, ESCALATES will produce a model that pools all measures and uses measure-specific fixed effects, which will allow for an assessment of an “overall” effect of the interventions.

#### Assessing contextual factors and heterogeneity of intervention effects

To assess heterogeneity of intervention effects, ESCALATES will identify factors associated with substantial improvement for some practices and lack of change or worsening performance for others. For instance, the ESCALATES team may observe that practices receiving the external support strategy of data feedback/benchmarking resulted in significantly improved ABCS performance measures over time as compared to a group of practices receiving expert consultation or a control group of practices. On further analysis of the effect of type of practice (moderator) on the observed association, however, the ESCALATES team may find that practices affiliated with large health systems showed greater improvements in ABCS measures than independent practices. Thus, this effect modification analysis is designed to capitalize on the large sample size and diversity of practices and several different intervention strategies that will be tested by Cooperatives to identify heterogeneity of ABCS measures and practice capacity measures of association across levels of modifying variables. Another key variable of this analysis involves the fidelity with which practices implement QI tools to address ABCS. Data on implementation fidelity will be collected using the external support/practice implementation tracking data. ESCALATES will create meaningful categories of this variable to include in the modification analysis, such as practices that implement QI tools consistently and to a high degree of fidelity will likely perform better at improving ABCS and practice capacity than those that do not, even when both groups of practices receive the same external support.

This analysis will extend the DID models proposed above by testing individual moderators with a three-way interaction of intervention group, time point and potential moderator. Our choice of variables to include will be based on theory and on triangulation with qualitative findings. Potential moderators include practice characteristics, as significantly associated with ABCS outcomes at baseline, and contextual factors that emerge during implementation. Moderators may be identified through surveys or qualitative data. Moderators identified through qualitative data will be quantified whenever possible. In addition to testing individual moderators, we will consider a composite moderator, which may more strongly moderate the effect of the intervention strategies on ABCS outcomes than any single moderator. Specifically, ESCALATES will create a combined moderator that is developed as a weighted combination of individual selected moderators to identify for which practices different intervention strategies may be preferred. Combining moderators has been shown to exhibit a larger moderator effect size than any individual moderator [67]. We will develop and evaluate a composite moderator using the parametric approach developed by Kraemer [69], which constructs a composite moderator from a weighted average of moderators that are weighted to maximize the correlation between the outcome difference between randomly paired practices and the average of the two values of the moderator variable.

In addition to these quantitative analyses of heterogeneity, we will also conduct qualitative analyses to evaluate the multilevel contextual factors that could affect either the interpretation of the results of each analysis, or that might be useful for others attempting to replicate or reinvent interventions in different times or situations [70, 71]. These analyses will use multiple data sources across multiple levels (from the practice and health care system, the intervention/evaluation team, and the local and national policy context), and will attempt to include diverse stakeholder perspectives of the most important contextual factors at critical points in the project/intervention, to help understand what happened here and why, and what someone else would need to know to transport/re-invent what was learned here in a different time and place. These analyses will enhance the external validity of ESCALATES findings.

#### Summative explanations for variation in outcomes across Cooperatives

To analyze why some intervention strategies are more effective than others, the ESCALATES team will analyze data among a group of high and low performing practices, using the same steps for qualitative data analysis described above. Data for this analysis will include fieldnotes, interviews, and external support/practice implementation tracking data which will allow us to establish what the practices implemented to improve ABCS delivery. Importantly, the qualitative team will be blinded to a practice’s status as a high or low performer during the data collection and analysis process. The qualitative team will identify mechanisms of practice change with regard to ABCS performance, the factors that influence a practice’s ability to implement or not implement change, and practice members’ experiences with their respective Cooperative’s intervention. At this stage, the qualitative team will bring in other data sources to make sense of the characteristics that distinguish practices. The end product will be a table of 40–60 practices and the factors or elements the qualitative team identifies as potentially important to practice change and improvement, and whether this element is present or absent for a practice. Next, the quantitative team will add practice performance to this table. A joint display will be created for a mixed methods analysis comparing the high and low performance practices in terms of qualitative findings and quantitative results. In order to distill a clear understanding of the factors associated with successful implementation, the qualitative team will use this information to conduct a deviant case analysis [72], examining in greater detail practices not conforming to our initial interpretation of what distinguishes low and high performing practices. As a final step, the full ESCALATES team will reflect on findings to fully identify the range of factors and characteristics vital to successful implementation of ABCS among which types of smaller practices and the key elements of external support required for practice improvement and for developing ongoing QI capacity.

### Trial status

The ESCALATES team has engaged the seven Cooperatives in the data harmonizing process, conducted one site visit with each Cooperative, and are working to refine a common set of measures and procedures for data collection. Cooperatives’ recruitment of practices is in progress. The start of baseline data collection and implementation of interventions will vary by Cooperative with start dates scheduled between January 2016 and April 2016.

## Discussion

### Significance

The EvidenceNOW Initiative is a unique national partnership between Cooperatives in seven distinct and diverse geographic regions of the USA who are each bringing together leaders and creating health care extension services with the credibility, expertise, relationships, and capacity to engage a large number of small- to medium-size primary care practices in rapid dissemination and implementation of CVD prevention guidelines. These types of primary care demonstration projects, pilot studies, and internally driven improvement efforts are typically not rigorously evaluated; when they are, they are not evaluated in a way that includes the evidence needed to know both *that* an innovation worked and *how* it worked so that lessons learned about implementation can be translated to other settings. The national-level ESCALATES evaluation addresses these gaps via an overarching mixed methods evaluation. Key elements of our evaluation are summarized in Table 2 above. Quantitative data will help us identify the most effective combinations of external support strategies for rapidly disseminating and implementing evidence into practice for various practice types, contexts, and organizational characteristics. Qualitative data from Cooperatives and selected practices will help us understand why and how those combinations are effective. At several points in the evaluation, these two databases will be merged to form a mixed methods analysis. The multiple data sources and perspectives that inform our evaluation, and our focus on contextual factors that explain cross-collaborative variations in ABCS and practice capacity will provide an unusually high level of external, as well as internal validity.

### Limitations

This protocol has several limitations. First, it may not be possible to harmonize measures across Cooperatives to the extent desired to optimize evaluation. This may be especially true for ABCS performance measures. Should this occur, the ESCALATES team has two strategies for addressing this limitation: (1) harmonize data for as many Cooperatives/practices as possible and conduct the analyses proposed on a subset of practices, and (2) treat each Cooperative as a case to conduct a comparative case analysis which uses Cooperative reported outcomes and synthesizes these findings with qualitative data. Second, some practices may be lost to follow-up for various reasons, while others may experience difficulties collecting study data as proposed. Such differential reasons for attrition may create biases in the sample. To mitigate this problem, the ESCALATES team will use a tracking mechanism to record practices that drop out and to identify practices that participate in the intervention but were unable to provide data. ESCALATES will follow-up with Cooperatives to identify the reasons data are not provided by practices. For practices that drop out, ESCALATES will have baseline demographic data (from the PS) and we will compare these practices with those remaining in the study to assess possible biases. Third, analysis of disparities is dependent on Cooperatives’ ability to extract consistent disparities data at the practice level from practices. The ESCALATES team will work with Cooperatives to extract EHR data to assess disparities, including race/ethnicity, age, and gender. ESCALATES will learn why these data are or are not able to be extracted, ESCALATES will understand how practices generated these data (e.g., patient report) and, if these data are not available, ESCALATES will ask participating practices to make estimates via the PS. Although these data may not be 100 % accurate, in the absence of better data, they can provide information on whether a practice has a predominant minority or a predominant Spanish-speaking patient panel, for example. The ESCALATES team can then use these data as a “crude” measure of disparity.

### Impact

A national effort of this scale and focus is unparalleled and offers a tremendous opportunity to learn how to build a national extension infrastructure and close the evidence-to-clinical practice gap among primary care practices. Learning evaluations such as ESCALATES, where innovators, evaluators, and funders work collaboratively and learn from each other, are critical to understanding the impact these transformative initiatives have on practice and patient outcomes [40]. The findings from this evaluation have the potential to inform the development of a national health care extension system in the USA, to contribute to what is known about how best to rapidly disseminate and implement evidence into primary care practices, and to contribute to a richer understanding of the complex set of practice organizational and cultural factors from which capacity and related quality-related outcomes emerge.

## Abbreviations

ABCS, aspirin prescribing, blood pressure and cholesterol control, and smoking cessation; AHRQ, Agency for Healthcare Research and Quality; BIC, Bayesian Information Criterion; CDC, Center for Disease Control and Prevention; CFIR, Consolidated Framework for Implementation Research; CMS, Medicare and Medicaid Services; CPCQ, change process capacity questionnaire; CVD, cardiovascular disease; D&I, dissemination and implementation; DHHS, Department of Health and Human Services; DID, differences-in-differences; EHR(s), electronic health record(s); ESCALATES, evaluating system change to advance learning and take evidence to scale; PCM, practice change model; PI, principal investigator; PMQ, practice member questionnaire; PS, practice survey; QI, quality improvement
